# Root phenotypes as modulators of microbial microhabitats

**DOI:** 10.3389/fpls.2022.1003868

**Published:** 2022-09-23

**Authors:** Henry W. G. Birt, Courtney L. Tharp, Gordon F. Custer, Francisco Dini-Andreote

**Affiliations:** Department of Plant Science and Huck Institutes of the Life Sciences, The Pennsylvania State University, University Park, PA, United States

**Keywords:** plant-microbe interaction, root anatomy, root architecture, traits, root exudates

## Abstract

Plant roots are colonized by a multitude of microbial taxa that dynamically influence plant health. Plant-microbe interactions at the root-soil interface occur at the micro-scale and are affected by variation in root phenotypes. Different root phenotypes can have distinct impacts on physical and chemical gradients at the root-soil interface, leading to heterogeneous microhabitats for microbial colonization. Microbes that influence plant physiology will establish across these heterogeneous microhabitats, and, therefore, exploiting variation in root phenotypes can allow for targeted manipulation of plant-associated microbes. In this mini-review, we discuss how changes in root anatomy and architecture can influence resource availability and the spatial configuration of microbial microhabitats. We then propose research priorities that integrate root phenotypes and microbial microhabitats for advancing the manipulation of root-associated microbiomes. We foresee the yet-unexplored potential to harness diverse root phenotypes as a new level of precision in microbiome management in plant-root systems.

## Introduction

Plant health is strongly influenced by microorganisms that live within or near plant tissues (Badri et al., 2013; Dini-Andreote, 2020; Fu et al., 2020). Many of these microbial modulators of plant health inhabit the rhizosphere – the narrow zone of soil adjacent to plant roots (Hiltner, 1904). The active manipulation of the rhizosphere microbiome can promote beneficial plant-microbe interactions to enhance agricultural sustainability, crop resilience, and yield (Trivedi et al., 2017). This could be achieved by altering abiotic conditions in the rhizosphere to direct the assembly and functioning of beneficial microbial groups. However, given the microscopic scale at which plant-microbe interactions take place, augmenting microhabitats *via* field-scale approaches (e.g., soil physical management and/or nutrient manipulation) can be a coarse approach for processes that operate on a much finer scale. Nevertheless, manipulating the soil environment at a scale meaningful to microbes is possible by focusing on root-soil interactions and the chemical gradients they create in the rhizosphere. Such gradients are formed *via* root exudates and sloughed-off cells, immobilization and mobilization of nutrients, and physical disruption of aggregates as roots grow through the soil matrix (for an in-depth review on rhizosphere gradients, please see Benard et al., 2019; Liu et al., 2022; and Stassen et al., 2021). These gradients are arise from complex interactions between the plant’s metabolism, root phenotypes, and soil characteristics – including the microbiome (Yan et al., 2004).

Root phenotypes are constituted of distinct phenes – i.e., traits under the plant genetic control (e.g., number of root cortical aerenchyma, or rooting angle) – that aggregate to form an overall phenotype (e.g., a steep, deep, and cheap root phenotype; York et al., 2013). As these vary across plant species and genotypes, and result in different conditions in the rhizosphere, root phenotypes can be targeted as subjects of breeding efforts to modulate microhabitats; however, plant breeding may have already ‘unintentionally’ selected diverse root phenes that influence microbial microhabitats (Lynch, 2019). For instance, barley cultivars with more root hairs resulted in reduced root-induced compaction in the rhizosphere (Koebernick et al., 2018). Therefore, explicitly considering the impact of specific phenes as modulators of microbial microhabitats can broaden our perspective on microbiome assembly and targeted management. This could attain outcomes not yet achievable by either a microbe-centric (e.g., microbial inoculation) or plant-centric (e.g., root nutrient acquisition without microbes) standpoints. To advance research efforts on this theme, here we discuss how root phenes and their combinations resulting in various root phenotypes affect the abiotic rhizosphere environment with consequences for microbiome assembly and functioning. Particularly, we explore variations in anatomical and architectural root traits that directly influence microhabitats in the rhizosphere. For example, *via* alterations in nutrient availability, or the extent and distribution of microhabitats through the soil matrix. Lastly, we provide a perspective on efforts that can facilitate the integration of root phenomics and microbial ecology, with the potential to produce a new level of precision in microbiome manipulation in the plant rhizosphere.

## Root architecture

Root architecture determines the spatial and temporal organization of root tissues through the soil matrix. The combination of phenes that give rise to a root architectural phenotype includes traits affecting root length, branching, angle, and the growth rate of both axial and lateral roots (York et al., 2013). As nutrients and water availability are not evenly distributed throughout the soil profile, root architecture influences the ability of plants to exploit these resources, with implications for plant health, status, and performance (Lynch, 2019). Root architecture also indirectly influences water and nutrient availability in microbial microhabitats (Jobbágy and Jackson, 2001; Nunes et al., 2020), with implications for microbiome assembly (Hartman and Tringe, 2019). In this section, we discuss aspects of root architecture based on root spatial distribution, as less is known about plant root temporal dynamics and their influence on the rhizosphere microbiome (Fierer et al., 2010); even though studies of rhizosphere dynamics through plant developmental stages, can represent indirect assessments of root temporal dynamics (Edwards et al., 2018; Dibner et al., 2021; Xiong et al., 2021).

### Rooting depth

**Figure 1:**
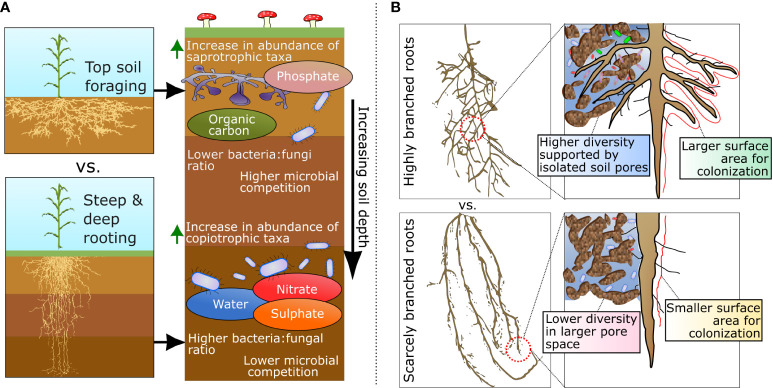
**(A)** Differences in root architecture based on the spatial distribution of roots through the soil profile and the differential distribution of microbial taxa and resources in the rhizosphere with increasing soil depth. **(B)** Variation in root branching and its influence on mediating the establishment and distribution of microhabitats in the rhizosphere.

Plant root depth is mostly determined by the angle and length of roots (Ramalingam et al., 2017), both of which can be affected by host genetics and plant-microbe interactions (e.g., *via* auxin production; Myo et al., 2019). Rooting depth has the potential to affect nutrient availability to microbes and thus their fitness (Pekkonen et al., 2013; Turlure et al., 2019) as varying root depths promote access to specific resource pools distributed across distinct soil horizons (**Figure 1**). For instance, less mobile soil resources such as phosphorous (P) and particulate organic matter are generally at greater concentrations at the top soil horizons, while nitrogen, sulfur, and sodium can easily move through the soil profile and accumulate at deep soil horizons (Jobbágy and Jackson, 2001; Nunes et al., 2020). This spatial distribution of resources also leads to a vertical stratification of microbiomes, with distinct taxonomic compositions and functional capabilities (Jiao et al., 2018). For example, P-solubilizing bacteria have been found to be significantly more abundant at shallower soil depths, where P tends to accumulate (Pastore et al., 2020). Therefore, by selecting phenes that create shallow rooting phenotypes, roots are in proximity to higher mineral P (Sun et al., 2018) and greater abundances of P-solubilizing bacterial taxa (Paul et al., 2018). Conversely, given the vertical distribution of soil taxa, deeper rooting plants can also be in association with different bacterial taxa. For example, members within the candidate phyla Dormibacteraeota have been shown to hold adaptations for surviving in deep soil horizons, including distinct metabolisms for carbohydrate storage, spore formation, and carbon monoxide oxidation (Brewer et al., 2019). As such, it is possible to speculate that exposure of plants to different microbial taxa in soil can result in variable effects on plant physiology and performance.

Deep rooting phenotypes can also indirectly influence water availability in the rhizosphere in arid environments, where water tends to become available in deeper soil layers (Broedel and Tomasella, 2017). Thus, allowing certain microbes to establish in the rhizosphere where water limitation may otherwise inhibit their persistence. This is particularly relevant because most soil microbes respond strongly to soil moisture variations (Neilson et al., 2017; De Vries et al., 2020). Water is necessary to microbes as a solvent for intracellular biochemical reactions, and its presence affects the mobility and access to nutrients (e.g., nitrogen, potassium, phosphorous), in addition to oxygen needed for aerobic growth (Tan et al., 2021). Furthermore, microbial cells are often in an osmotic balance with their immediate surroundings *via* the accumulation of compatible solutes/osmoprotectants (e.g., betaines) to maintain water potential in their favor under drier conditions, which incurs metabolic costs to the cell (Schimel, 2018).

Variation in rooting depth has the potential to affect the balance of ecological interactions between groups of microbes in the soil profile. For instance, decreases in fungal to bacterial ratios have been observed in the rhizosphere of plants with longer specific root lengths (Demenois et al., 2018; Wan et al., 2021), a common phenotype selected in breeding programs (Hund et al., 2009; Lopes and Reynolds, 2010; Henry et al., 2011). This occurs because at deeper soil layers, fast-growing copiotrophic bacteria can outcompete slower-growing fungi for access to the labile carbon released through rhizodeposits (**Figure 1**). Thus, causing a reduction in fungal biomass as other forms of more recalcitrant carbon – more efficiently processed by saprophytic fungi – are locally absent or present in shorter supply (Jobbágy and Jackson, 2001; Rousk and Bååth, 2011). However, at shallower soil depths, fungi can often – or at least partially – outcompete some bacterial taxa. This occurs due to the capacity of most saprotrophic fungi to process recalcitrant forms of carbon more efficiently (e.g., lignin, pectin, glycans) that, in general, bacteria lack the enzymatic capacity or take longer to degrade and utilize (Janusz et al., 2017).

### Root branching

Plant-root branching patterns are formed through the development of lateral roots, which influence the total surface area of the root that is in contact with the soil (Liu et al., 2014). This alters the total size of the rhizosphere that is available for microbial colonization (**Figure 1**). Through plant development, root branching often causes narrower roots to be produced at lower order roots, thus allowing the exploitation of smaller soil pores and/or micro-aggregates (Jungk, 2001). Within the context of the rhizosphere, the size, distribution, and connectivity of soil pores are important determinants of the formation of microbial microhabitats (Kim et al., 2022; Vogel et al., 2022). In this scenario, it is possible that smaller pores act as isolated habitat patches, which can influence competitive interactions and strengthen patterns of coexistence among microbial taxa (Lowery and Ursell, 2019). This is corroborated by the fact that finer roots have been shown to contain more diverse microbial communities when compared to higher-order roots (Saleem et al., 2016; Pervaiz et al., 2020; King et al., 2021; Luo et al., 2021). Moreover, some microbial taxa have also been shown be more abundant in specific root orders (Wang et al., 2017), possibly due to their ability to compete in distinct habitat patch sizes with characteristic biotic and abiotic conditions. For example, microbial taxa affiliated with Oxalobacteraceae, Comamonadaceae, and Polyangiaceae were shown to mostly colonize either the nodal or seminal roots of *Brachypodium distachyon* (Kawasaki et al., 2016). In line with that, members of Oxalobacteraceae have also been shown to be able to restore lateral root development in defective mutants once the soil had been conditioned by plant flavones (Yu et al., 2021). Interestingly, this finding also indicates that lateral root development can – in some cases – in part be determined by the feedback between plants and their associated microbes in the rhizosphere.

## Root anatomy

Root anatomy is determined by the cellular composition and function of the structures that make up plant roots (e.g., cortical tissue, vascular bundles, root hairs, and root cap). The arrangement and chemical composition of tissues and cells within the root directly affect the physical environment of the soil (Kawa and Brady, 2022). This occurs *via* changes in the rates of diffusion of nutrients and sloughed-off cells at the root-soil interface, the indirect effects on gradients of water and exudates, as well as the modulation of chemical properties (e.g., oxygen diffusion, pH) of the rhizosphere at the micro-scale (Kirk et al., 2019; Salas-González et al., 2021).

### Root hairs

Root hairs are single-celled outgrowths from the root epidermis which extend the plant’s root surface area, increase nutrient and water uptake (Qiao and Libault, 2013). Root hairs increase the enzymatic activity and the extent of the rhizosphere, depending on their length and density (Ma et al., 2018). Such traits help plants to tolerate drought and accumulate phosphorous (Marin et al., 2021). In addition, the area of soil in contact with root hairs can serve as a zone of carbon accumulation, which has the potential to enrich microbial activity and diversity (Kravchenko et al., 2019). For example, hairless barley mutants were shown to result in differences in rhizosphere microbiome composition and lower community diversity (Robertson-Albertyn et al., 2017). In another study, a collection of bacterial taxa isolated from the root hairs of *Leersia oryzoides*, and inoculated in rice (*Oryza sativa*, a domesticated relative), were shown to stimulate root hair formation (Verma et al., 2018); these same bacterial taxa were also able to protect the plant against fungal pathogens. Together, these studies indicate the beneficial impacts of specific microbial taxa on dynamically influencing both root phenotypes and plant health.

Altering root hair characteristics could actively augment soil pore space and aeration. For example, in barley, genotypes with more root hairs resulted in altered soil porosity and connectivity, and higher pore volume which alleviated root-induced soil compaction. (Koebernick et al., 2017). The physical and chemical consequences of soil compaction can have a direct effect on the soil microbiome, for example, by favoring methanogenic and denitrifier taxa under oxygen-limiting conditions (Weisskopf et al., 2010). Additionally, root hairs can increase root exudation (Holz et al., 2018) due to passive transport being easier through root hairs compared to other root orders. This occurs due to a large surface area of contact with soil, and a lack of lignification and suberization in root hairs (Boyer and Kramer, 1995). Despite the direct effect of root exudation on microbial diversity, activity, and biomass (Eisenhauer et al., 2017), greater root hairs can – in some cases – also result in lower microbial activity in the rhizosphere. For example, genotypes with abundant root hairs can more efficiently uptake water in the root-soil interface (Segal et al., 2008), thus limiting its availability for microbial growth and activity in the rhizosphere (Manzoni et al., 2016).

### Root cortical aerenchyma

**Figure 2:**
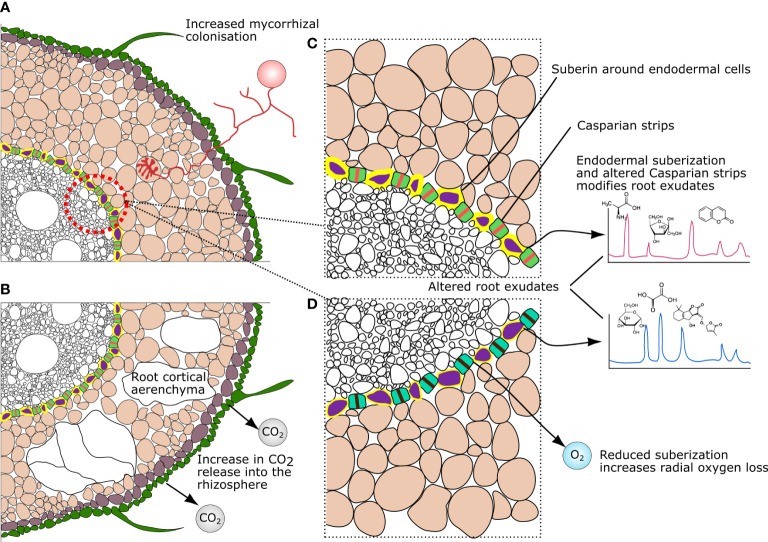
Variation in root anatomical traits and their influences on chemical gradients modulating microbial microhabitats in the rhizosphere: **(A, B)** variation in root cortical aerenchyma; and **(C, D)** Suberin and Casparian strip modification.

Variation in root cortical aerenchyma (RCA) across plant species and genotypes can also directly influence the rhizosphere microbiome (**Figure 2**). For example, greater occurrence of RCA in the cortex results in a lower number of cortical cells available for mycorrhizal root colonization (Dreyer et al., 2010; Galindo-Castañeda et al., 2019). This can favor lower carbon allocation in the root cortex, but disfavor mycorrhizal plants, which rely on efficient symbiosis for survival/performance under phosphorous and water-limited conditions (Begum et al., 2019). Moreover, the presence, abundance, and distribution of RCA affect the rates of nutrient and gas exchange in the rhizosphere (Smits et al., 1990; Hu et al., 2014). For example, increases in the abundance of RCA in maize roots was shown to reduce phosphate exudation (Hu et al., 2014), which can – in turn – influence the composition of bacterial and fungal communities (Finkel et al., 2019; Gumiere et al., 2019). In another study, RCA was shown to affect carbon dioxide (CO_2_) exchange in the root zone, directly affecting gradients of pH at the root-soil interface *via* carbonic acid production (Kirk et al., 2019). In fact, local pH might be one of the most important factors influencing the rhizosphere microbiome (Fierer and Jackson, 2006; Malik et al., 2017; Bahram et al., 2018). In particular, pH is known to affect microbial communities by causing variation in nutrient availability and uptake (Peterson, 1982), and by influencing the kinetics of biochemical reactions (e.g., nitration; Van Hulle et al., 2007).

### Root endodermal barriers

Suberin deposits and Casparian strips are the main endodermal structures regulating the ionic flow from the root (Beck, 2005). Modifications in suberin deposition and Casparian strip permeability alter the release of metabolites in the rhizosphere (i.e., root exudates) (Calvo-Polanco et al., 2021). This has been shown to influence the abundance of a range of microbes, including Pseudomonadaceae (Durr et al., 2021; Salas-González et al., 2021) – a bacterial family displaying functions associated with plant health (e.g., disease suppression, plant-growth promotion) (Preston, 2004). In addition, endodermal suberin is degraded by the plant in response to ethylene (Barberon et al., 2016) – a plant hormone that is modulated and able to be produced by specific rhizosphere microbes (Ravanbakhsh et al., 2018) – as well as by plant-microbe feedback *via* abscisic acid (Salas-González et al., 2021). Therefore, suberization can potentially be modulated *via* plant-microbe feedback by diverse pathways. This has implications for the plant’s ionic homeostasis as root endodermal barriers also influence the flow of ions, such as iron and salt, into root tissues (Durr et al., 2021; Salas-González et al., 2021).

Suberization of endodermal cells also alters the radial oxygen loss into the rhizosphere (Armstrong and Armstrong, 2005). This effect could also modulate differential responses of microbial taxa, as oxygen can rapidly be consumed by aerobic copiotroph taxa (Hojberg and Sorensen, 1993), and a range of rhizosphere microbes have shown varying abilities to adapt to shifting oxygen conditions (Lecomte et al., 2018). Moreover, as suberization also affects the thickness and rigidity of the cortex, suberin deposition has often been selected in breeding programs to promote resistance against pathogens (Valenzuela-Estrada et al., 2012). In this case, by conferring a physical barrier to pathogen penetration. However, these breeding lines may find new uses in influencing the overall microbiome given the newly discovered role of suberization in rhizosphere microhabitat manipulation.

## Research priorities for integrating root phenomics and microbial ecology

Identifying root phenes for targeted manipulation of rhizosphere microbiomes will require coordinated research efforts from microbial ecologists, plant scientists, and plant breeders. To stimulate these efforts, we suggest research priorities to gain a better understanding of how various root phenotypes modulate microbial microhabitats in the rhizosphere:

- Gene mutants and recombinant inbred lines can be utilized to isolate phene level differences in roots that influence microbial microhabitat and – in turn – microbial diversity and function (Prokhnevsky et al., 2008; Song et al., 2016; Polania et al., 2017). Nevertheless, these mechanisms must be contextualized within edaphic conditions, disease, climatic factors, and plant developmental stage, all of which have been demonstrated to influence plant-associated microbiomes (Chaparro et al., 2012; Jansson and Hofmockel, 2020; Gao et al., 2021; Bell et al., 2022).
- Root phenotypes have been shown to differ between domesticated crops and their wild relatives (Martín-Robles et al., 2018). As such, the integration of microbial ecology and root phenomics needs to consider ancestral and domesticated crops to gain a broader understanding of phene diversity influencing microbial selection in the rhizosphere (Pérez-Jaramillo et al., 2018).
- Plant-microbe interactions take place at micro-scales. Describing this system will require methods capable of characterizing fine-scale changes in the rhizosphere. For example, the use of matrix-assisted laser desorption/ionization-mass spectrometry imaging (MALDI-MSI) to monitor metabolite release (Korenblum et al., 2020), lux biosensors to identify the sites of colonization of microbes in response to specific root metabolites (Pini et al., 2017; Geddes et al., 2019), and par-seqFISH (parallel sequential fluorescence *in situ* hybridization) to provide cell-level transcriptional responses of microbes within microhabitats (Dar et al., 2021).

## Conclusion

Here, we reviewed how differences in root phenotypes can influence resource availability and the spatial configuration of microbial microhabitats in the rhizosphere. We also outlined research priorities for integrating root phenomics with microbial ecology to manipulate microhabitats at the root-soil interface. We argue that modifying microbial microhabitat *via* root phenotypes could provide an unprecedented level of control on plant-associated microbes. Advancing research that effectively promotes the manipulation of microbiomes in plant roots could increase agricultural sustainability, yield, and resilience in the face of the rising food demand and an increasingly less stable climate.

## Author contributions

HB, CT, GC, and FD-A wrote, discussed, and reviewed the manuscript. All authors contributed to the article and approved the final version.

## Acknowledgments

This work was support by the USDA-NIFA Award 2021-67013-33723 and the Fulbright Lloyd's Award provided by the US-UK Fulbright Commission.

## Conflict of interest

The authors declare that the research was conducted in the absence of any commercial or financial relationships that could be construed as a potential conflict of interest.

## Publisher’s note

All claims expressed in this article are solely those of the authors and do not necessarily represent those of their affiliated organizations, or those of the publisher, the editors and the reviewers. Any product that may be evaluated in this article, or claim that may be made by its manufacturer, is not guaranteed or endorsed by the publisher.
